# Greenhouse Gas Emissions From Biofilters for Composting Exhaust Ammonia Removal

**DOI:** 10.3389/fbioe.2022.918365

**Published:** 2022-06-15

**Authors:** Bin Shang, Tanlong Zhou, Xiuping Tao, Yongxing Chen

**Affiliations:** Key Laboratory of Energy Conservation and Waste Management of Agricultural Structures, MARA, Institute of Environment and Sustainable Development in Agriculture, Chinese Academy of Agricultural Sciences, Beijing, China

**Keywords:** empty bed retention time, ammonia biofilter, greenhouse gases, nitrous oxide, waste management

## Abstract

Emissions of odorous compounds, such as ammonia (NH_3_), from composting have negative agronomic and environmental impacts. A biofilter is widely used for NH_3_ removal, with one of its potential detrimental by-products being nitrous oxide (N_2_O), which is a higher warming potential greenhouse gas (GHG). The aim of the study was to evaluate the effect of empty bed retention time (EBRT) on GHG emissions from biofilters for removing NH_3_ from composting. Composting experimental trials lasted 6 weeks, and composting materials were mixtures of dead pigs and manure. Three groups of biofilters with 1.2 m-height, 0.3 m-inner diameter, and 1.0 m media depth were conducted with EBRT of 30, 60, and 100s, respectively. Each treatment was performed in triplicate, and the gas was monitored using the dynamic emission vessel method. The Spearman’s correlation analysis showed a significantly positive correlation between inlet concentrations (ICs) of NH_3_ and increased N_2_O concentrations: ρ = 0.707, 0.762, and 0.607 with *p* ≤ 0.0001 for biofilters with EBRT of 30, 60, and 100s, respectively. The fraction of NH_3_-N denitrified into N_2_O-N in biofilters with EBRT of 60 and 100s was higher than that with EBRT of 30s. The total global warming potential (GWP) increased by 126%, 162%, and 144% for biofilters with EBRT of 30, 60, and 100s, respectively. These results indicated that biofilters with longer EBRT will lead to higher GWP production. Future research on odorous mitigation for composting with biofilters should focus more on greenhouse gas emissions.

## Introduction

Composting has been applied worldwide as an environmentally friendly and cost-effective method for sanitation and recycling animal waste (Loyon, 2017; Zheng et al., 2020). However, one major complication during composting is the odor emission (Cheng and Hu, 2010; Huang et al., 2012; Paulot, et al., 2014), which not only poses a problem to the general public and environmental health but also causes adverse effects on vegetation surrounding the composting plants (Han et al., 2019). Ammonia (NH_3_) is considered the major contributor to odor from composting (Zhang et al., 2016; Zhu et al., 2016), and the precursors of particulate matter can be produced by the reactions of NH_3_ with sulfuric and nitric acid aerosols (Pinder, et al., 2007; Renner and Wolke, 2010; Kong et al., 2020).

Biofilters are widely used to reduce NH_3_ emissions from composting (Turan et al., 2009; Mudliar et al., 2010; Janni et al., 2014). Many studies have focused on the physical, chemical, and biological parameters influencing the biofiltration process (Park, et al., 2002; Yuan et al., 2019). A number of studies have shown that nitrous oxide (N_2_O) generation in biofilters is often accompanied by NH_3_ removal (Maia et al., 2012b; Yang et al., 2014a; Kong et al., 2020). Akdeniz and Janni (2012) observed that N_2_O generation ranged from −29.2% to 4.0% for a flat-bed biofilter with an empty bed retention time (EBRT) of 5s. Clemens and Cuhls (2003) observed that approximately 26% of NH_3_-N entered in the biofilters is converted into N_2_O-N. N_2_O emissions from biofilters can be affected by several factors, such as inlet NH_3_ concentration, temperature, moisture content, and pH value (Maia et al., 2012b; Yang et al., 2014a; Yang et al., 2014b; Dumont et al., 2014). High moisture content can cause regional anaerobic zones and increase the microbial activity, which favors N_2_O emission (Yang et al., 2014a). Compared to a high pH value (8.0–9.5), a low pH value (4.5–6.0) of the media can inhibit the generation of N_2_O reductase and reduce the emission of N_2_O (Yang et al., 2014b). N_2_O from biofilters was correlated significantly with the NH_3_ in the biofilters (Clemens and Cuhls, 2003). EBRT can affect microorganism absorption and the conversion process of NH_3_ in the biofilters (Shang et al., 2020). Shorter EBRT means faster gas flow velocity, which can change the oxygen (O_2_) gradients in the biofilter media and can lead to changes in denitrification (Maia, et al., 2012b). In addition, higher air flow can increase the emission rate of gas from composting. EBRT is one of the key parameters of biofilters for NH_3_ removal (Liu et al., 2017); however, few studies have investigated the effects of EBRT on emissions of N_2_O. A better understanding of the effects of EBRT on the generation and emission of N_2_O needs to be elucidated. In this study, the effects of EBRT on greenhouse gas (N_2_O and methane, CH_4_) emissions from pilot-scale biofilter systems were studied. The results can provide a promising tool for greenhouse gas reduction from full-scale biofilters.

CH_4_ is another important greenhouse gas emitted during composting (Zhu-Barker, et al., 2017). Biofilters also can be used to reduce CH_4_ emissions (Haubrichs and Widmann, 2006). Many studies performed on CH_4_ biofiltration utilized an EBRT of at least 4 min (La et al., 2018), which posed obstacles to the biofilter application. Little information is available in the literature about CH_4_ reduction capabilities during NH_3_ biofiltration.

Global warming potential for N_2_O and CH_4_ is 296 and 23 times higher than that of carbon dioxide (CO_2_), respectively (Pachauri et al., 2014). It is very important to examine the generation of greenhouse gas due to odor treatment by biofilters. Thus, the objectives of this study were 1) to investigate the emissions of N_2_O from biofilters with different EBRT and 2) to assess the greenhouse gas (N_2_O and CH_4_) emissions from biofilters for composting NH_3_ removal.

## Materials and Methods

### Experiment Materials

This study was conducted in the Beijng Anding pig farm, located in Daxing District, Beijing, China (39°62′N, 116°50′E). The composting materials and composting process have been described by Shang et al. (2020).

The mature compost, composted for about 4 months, was used as the medium material. Before the experiment, the mature compost was inoculated with activated sludge from the aerobic fermentation plant for wastewater treatment in the pig farm, and the water content of packing materials at the beginning of composting was adjusted to 57.4 ± 2.5%, according to Akdeniz and Janni (2012). The mature compost used for biofilter media had total carbon (C) and nitrogen (N) contents of 30.8 ± 2.8% and 2.9 ± 0.7%, respectively, with a pH of 7.0.

### Biofilter Design and Operations, Gas Sampling, and Analytical Method

**FIGURE 1:**
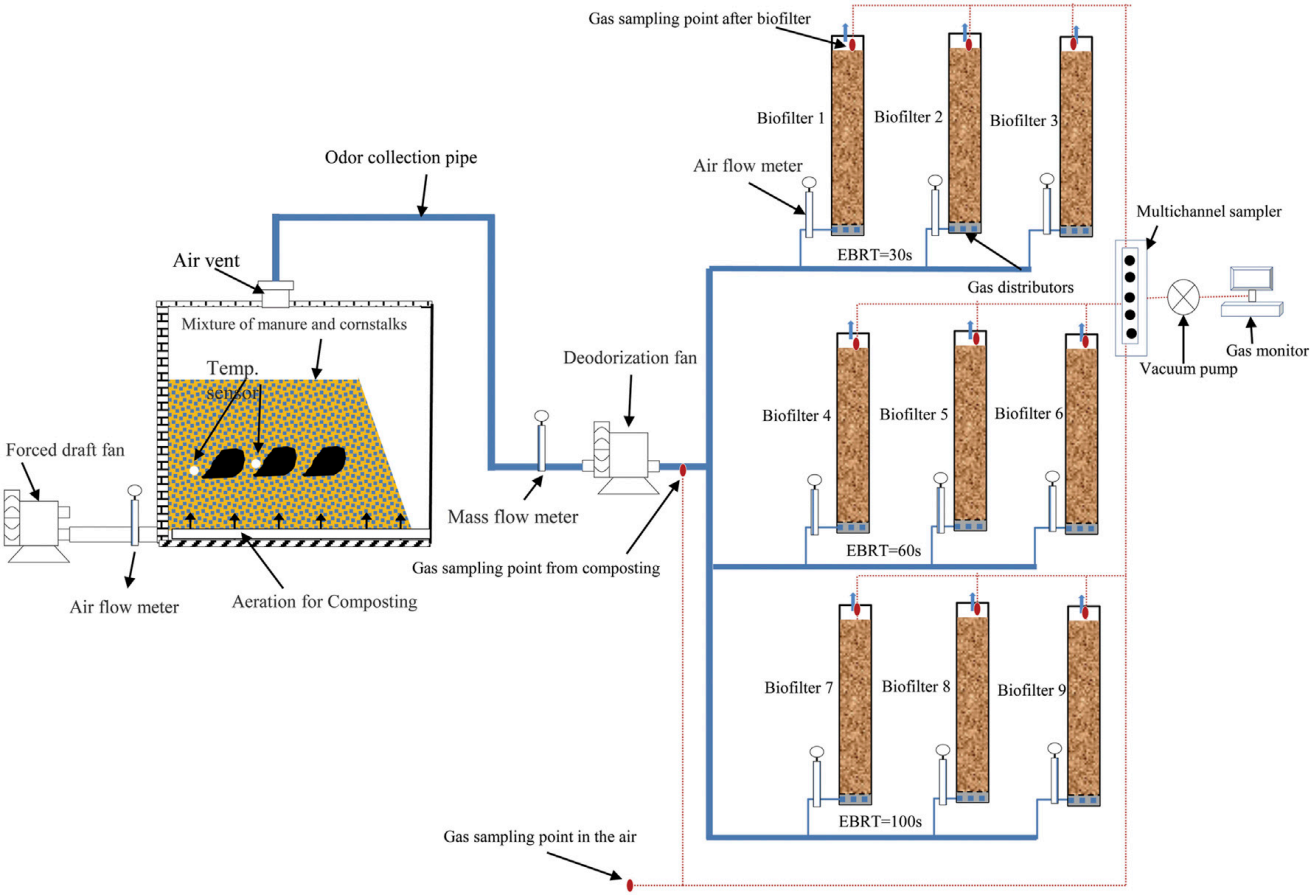
Schematic representation of the composting and the biofiltration system.

The biofilters were constructed with circular unplasticized polyvinyl chloride (UPVC) pipes. The dimensions were 1.2 m (height) and 0.3 m (inner diameter), with a 1.0 m depth of media (corresponding to a bed material volume *V* = 0.07 m^3^). Nine biofilters were divided into three treatments, and different EBRT (30s, 60s, and 100s) were set according to previous studies (Pagans et al., 2005). Each treatment was conducted with three replicates. EBRT = *V/Q*, where *Q* is the air flow rate in m^3^·s^−1^. The experimental arrangements are shown in Table 1. NH_3_, CH_4_, and N_2_O concentrations of air outside (1 sampling point), a gas inlet of biofilters (3 sampling points), and a gas outlet of biofilters (9 sampling points) were measured continuously and simultaneously using a photoacoustic multigas analyzer (model Innova 1412i, LumaSense Technologies, Ballerup, Denmark) every day. The deodorization system was constructed, as previously described (Shang et al., 2020), and the schematic is shown in Figure 1. The experiment corresponding to the composting period was carried out for 42 days. During the experiment, no water was supplied to the media of biofilters.

**TABLE 1:** Experimental performance of biofilters.

Treatment	Biofilter	Volume per biofilter (m^3^)	Empty bed retention time (EBRT, s)
1	1,2, and 3	0.071	30
2	4,5, and 6	0.071	60
3	7,8, and 9	0.071	100

### Data Analyses

All data were analyzed by Microsoft Excel 2016 and SPSS 22. The variables were tested for significance using Spearman’s correlation coefficient. The rations of gas concentrations in outlet and inlet biofilters were calculated (Dumont, 2018). A ration >1 indicates gas formation in the biofilter, and a ration <1 indicates gas depletion in the biofilters. The *t*-test (one side *p* < 0.05) was used to determine if there was a significant deviation from 1.

### Global Warming Potential Calculations

Global warming potential (GWP) was quantified as CO_2_ equivalent with a 100-year timescale: 1 kg CH_4_ and N_2_O emitted are equivalent to 23 and 298 kg CO_2_, respectively (Pachauri et al., 2014). The CH_4_, N_2_O, and GHG emissions (in kg eqCO_2_) were calculated by multiplying the aerobic rates, the concentration, and the GWP factor during the whole experiment which lasted for 42 days. In this study, the GWP of CO_2_ was not included because it is not considered GHG of agriculture (Buendia et al., 2019).

## Results and Discussion

### Emission of Nitrous Oxide From Biofilters

**FIGURE 2:**
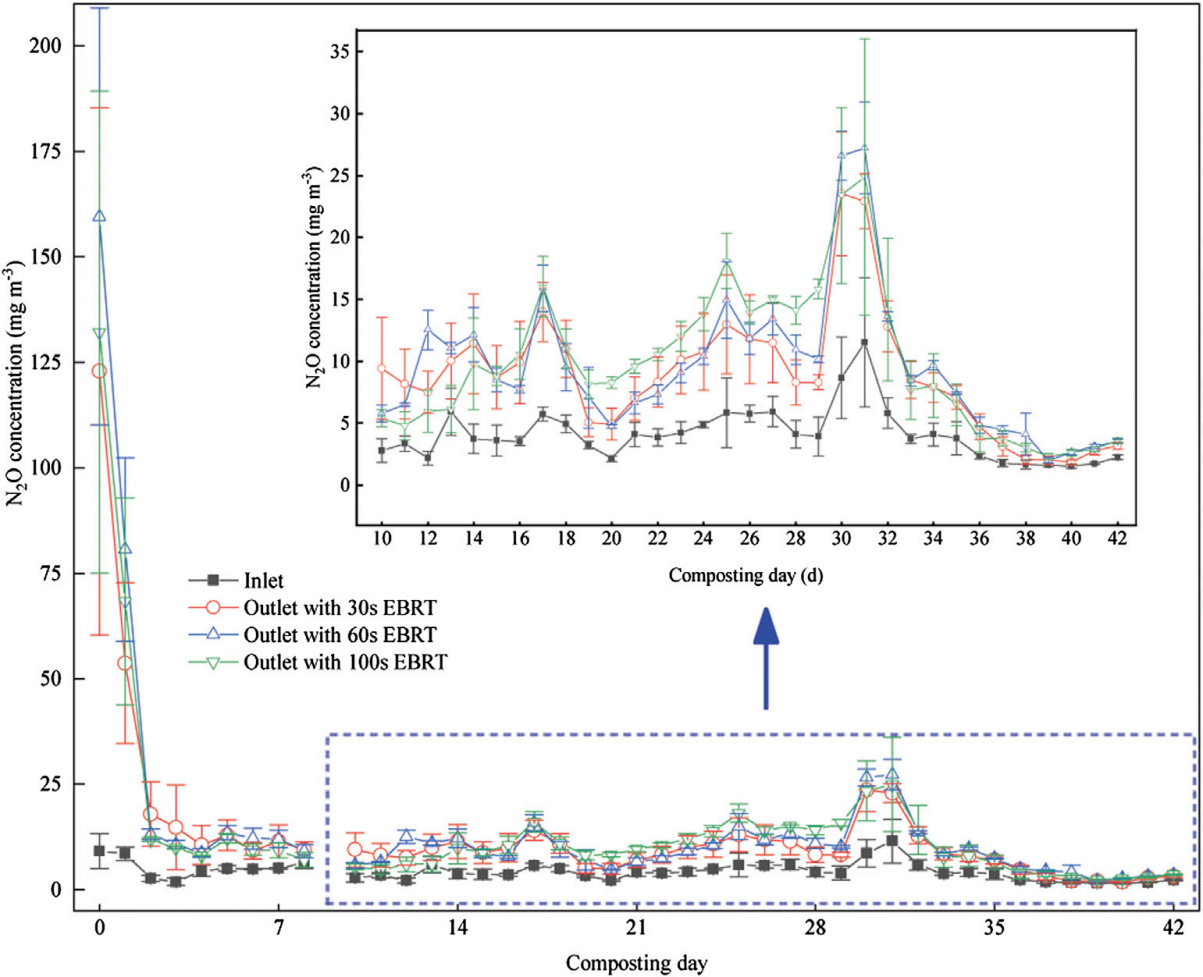
Daily N_2_O concentrations (mean ± SE) at the biofilter inlet (IC) and outlet (OC).

**FIGURE 3:**
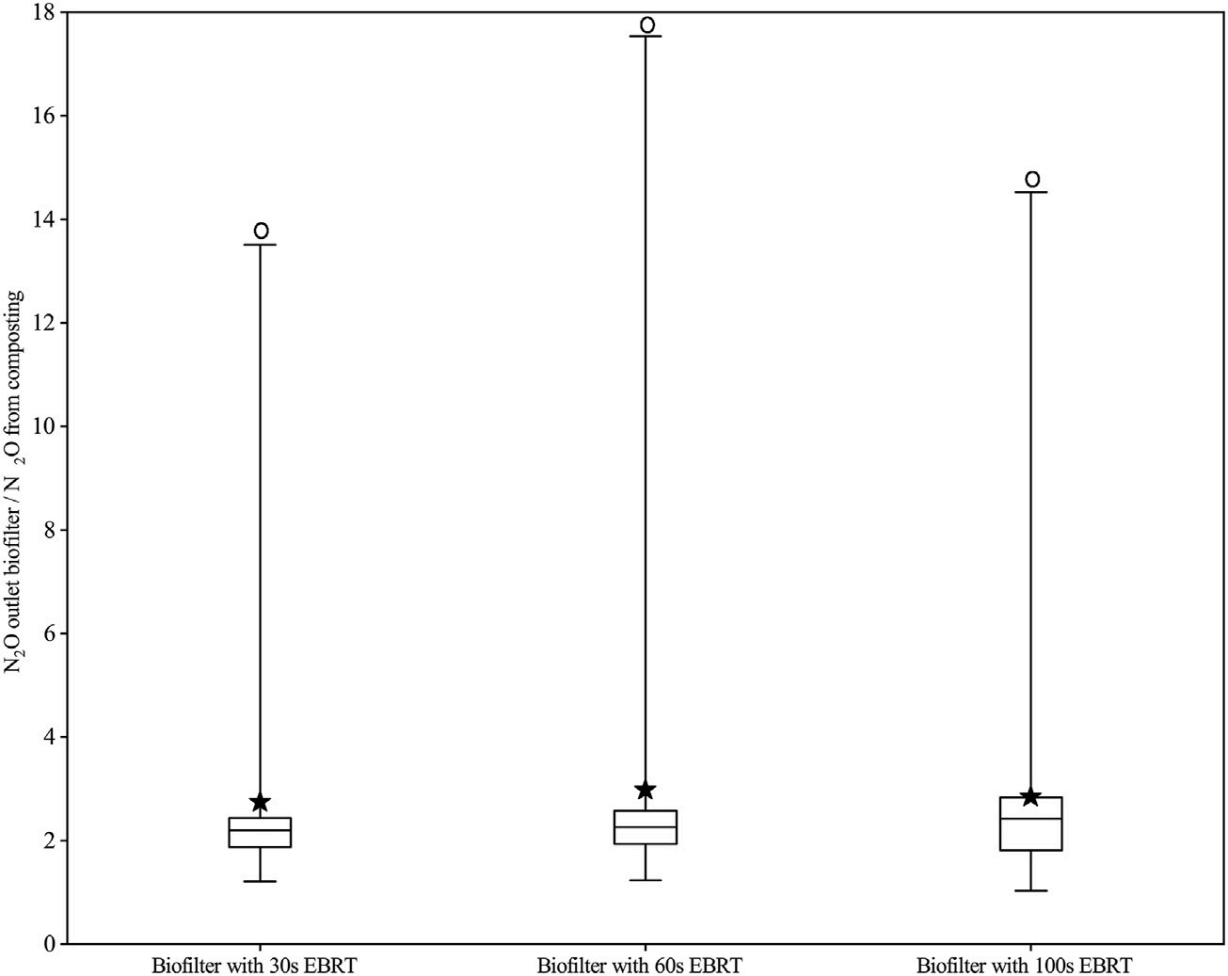
Boxplot: Ratio of N_2_O concentration of the outlet and inlet at different biofilters (Box border means 25 and 75% percentile, the solid line in the box means the median value, the solid pentacle means the mean value, whisker means the maximum and minimum values, and the white circle means significant differences between the inlet and outlet).

NH_3_, N_2_O, and CH_4_ concentrations in the air during the composting period were 5.6 ± 2.5, 1.0 ± 1.6, and 3.2 ± 1.6 mg m^−3^, respectively. The NH_3_ removal efficiencies have been described by Shang et al. (2020). The daily mean concentration of N_2_O from composting ranged between 1.5 and 11.5 mg m^−3^, while the daily mean N_2_O concentrations from biofilters were about around 1.0 mg m^−3^ (Figure 2). The large variation in N_2_O production was due to the different concentrations of NH_3_ and N_2_O from composting. The N_2_O concentrations at the biofilters’ outlet were in the range of 20%–1250%, 23%–1652%, and 3–1352% higher than those at the biofilters’ inlet for EBRT of 30, 60, and 100s. The outlet concentrations of N_2_O of biofilters with EBRT of 100 and 60s were higher than those with EBRT of 30s (Figure 3). But there are no significant differences between the biofilters with different EBRT.

With biofiltration systems, about half of the inlet NH_3_ is converted to nitrites or nitrates, and the other half is absorbed into the water as ammonium (Ottosen et al., 2011; Yasuda et al., 2017). Both ammonia oxidizer and denitrifier can produce N_2_O in a biofilter, but the majority of N_2_O was generated from denitrification (Kong et al., 2020). Maia et al. (2012a) pointed that higher EBRT may result in non-uniform oxygen distribution, which favors conditions for denitrification to generate N_2_O. In this study, the higher EBRT means a slower air flow rate, and the increasing N_2_O concentration from biofilters with increasing EBRT may result from more denitrification. In addition, when the air continuously goes through media in the biofilter, the higher EBRT implies that there is more content time of air and media, which can lead to high NH_3_-sorption, which favors the occurrence of nitrification–denitrification (Maia et al., 2012a). In this study, N_2_O concentrations were found to be higher, up to 16 times, than inlet concentrations, which may be due to the longer EBRT and higher inlet concentrations of NH_3_ (Dumont et al., 2014; Liu et al., 2017).

The inlet concentrations, inlet loads, and elimination capacities of NH_3_ may affect N_2_O generation. The Spearman’s correlation analysis showed a strong positive correlation between inlet NH_3_ concentrations and increased N_2_O concentrations: ρ = 0.707, 0.762, and 0.607 with (*p* ≤ 0.0001) for EBRT of 30, 60s and 100s, respectively. Inlet loads of NH_3_ and increased N_2_O concentrations were positively correlated: ρ = 0.685, 0.750, and 0.579 (*p* ≤ 0.0001) for EBRT of 30, 60s, and 100s, respectively. Elimination capacities of NH_3_ and increased N_2_O concentrations were also positively correlated: ρ = 0.706, 0.761, and 0.602 with *p* ≤ 0.0001 for EBRT 30, 60, and 100s, respectively. In this study, the inlet NH_3_ concentrations were between 12 and 447 mg m^−3^ due to different composting processes, while the overall removal efficiencies were 85.4%, 88.7%, and 89.0% for EBRTs of 30, 60, and 100s, respectively. The elimination capacities of NH_3_ have been discussed by Shang et al. (2020). NH_3_ removal was considered the net source of N_2_O in biofilters (Maia et al., 2012a). N_2_O is considered normal at biofilters treating NH_3_-containing air, being a byproduct of nitrification and denitrification, and the pathways of N_2_O formation reported in the literature, however, are complex. Kong et al. (2020) found that the inlet NH_3_ concentration can affect the nitrification process and the substrate availability for denitrification. In the present study, high inlet loads of NH_3_ promoted N_2_O generation, which were consistent with the other study (Kong, et al., 2020); the denitrification is the main pathway for N_2_O formation.

### Fraction of NH_3_-N Denitrified Into N_2_O-N

In the present study, there were significant differences in N_2_O emissions between biofilters with different EBRT due to different inlet loads of NH_3_ resulting from different EBRT. The fractions of NH_3_-N converted into N_2_O-N were 4.6%, 5.6%, and 5.1% for biofilters with EBRT of 30, 60, and 100s, respectively. The results were consistent with other studies (Table 2). Denitrification is considered the main source of N_2_O emission while nitrification is a trigger (Yang et al., 2014a; Dumont et al., 2014; Kong et al., 2020), and the presence of oxygen can inhibit the denitrification process. In this study, low EBRT means high gas flow rate, which results in more oxygen penetration into the media of the biofilter, further reducing the denitrification rates. Except for the EBRT, some other factors, such as the kind of media and moisture content may also affect N_2_O generation in biofilters. In Maia et al. (2012a), the compost of horse manure, cattle manure, chicken waste, woodchips, sawdust, and other materials were used as media for NH_3_ removal, fraction of NH_3_-N denitrified into N_2_O-N was 14% and 19% for one biofilter and two other biofilters, respectively. The results of Yasuda et al. (2009) confirmed that NH_3_ could be treated by biofilter with rockwool mixture without an extra increase of N_2_O. The percentage of N_2_O reduction efficiency ranging from 0.13 to 0.73% was found by Akdeniz (et al., 2011), who studied the removal of NH_3_ and N_2_O using biofilters with lava rock as media at 5s EBRT. The part of NH_3_-N converted into N_2_O was estimated to range from 10% to 40% (Dumont et al., 2014). Yang et al. (2014a) found that there was a slight increase in N_2_O when the media moisture content increased from 35 to 55%, but further increasing the moisture content to 63% triggered N_2_O generation rapidly. Yasuda et al. (2009) observed a higher N_2_O generation when the moisture content ranged from 65% to 52%; when the moisture content decreased to 48%, the N_2_O net generation decreased to nearly zero; when the moisture content decreased from 44% to 13%, N_2_O generation decreased. A moisture content of about 50% is recommended for efficient NH_3_ removal and less N_2_O generation. Yang et al. (2014b) showed that N_2_O concentrations ranged from 0.1 to 0.4 ppm with a pH of 8.0, and the acidified biofilters showed higher N_2_O concentrations than alkalized biofilters. In the present study, the values of the moisture content and pH of media change very little through the experiment. During the experiment, the pH of media was maintained at around seven for all biofilters, while the average moisture contents of media (mean ± SE, *n* = 12) were 48.3 ± 0.4, 48.5 ± 0.5, and 49.1 ± 0.4 for the biofilters with EBRT of 30, 60, and 100s, respectively.

**TABLE 2:** Literature overview of N_2_O generations in biofilters.

Biofilter media material	Empty bed retention time (s)	Experiment periods (d)	Inlet NH_3_ load (g.m^−3^.h^−1^)	Inlet NH_3_ concentration (mg.m^−3^)	N_2_O-N emission (g.m^−3^.h^−1^)	Inlet NH_3_-N to N_2_O-N (%)	Reference
Woodchips inoculated with activated sludge	12	124	2.4–3.0	8–12	Maximum was around 1	10–40	Dumont, et al. (2014)
Compost (mixture of horse manure, cattle manure, chicken waste, woodchips, sawdust, and others)	25	100	0.99	17.5	0.2	/^a^	Maia, et al. (2012b)
Compost (mixture of horse manure, cattle manure, chicken waste, woodchips, sawdust, and others)	20	21	0.47	11.2	/	14–19	Maia, et al. (2012a)
Mixture of wood chip and compost	34	22–35	5.24	31	/	1.9–2.3	Yang, et al. (2014a)
Mixture of pine wood chips and peat soil	42	/	1.5–3	13.7–26.6	/	5.2–14.8	Kong, et al. (2020)
Woodchips	1.4–3.3	/	8.7–67	19–86	0.2–0.5	1.3–21	Melse and Hol, (2017)
Mature compost	30–100	42	0.5–53.6	13–447	0.3–1.6	4.6–5.6	The present study

a “/” means no data.

### Emissions of CH_4_ From Biofilters

**FIGURE 4:**
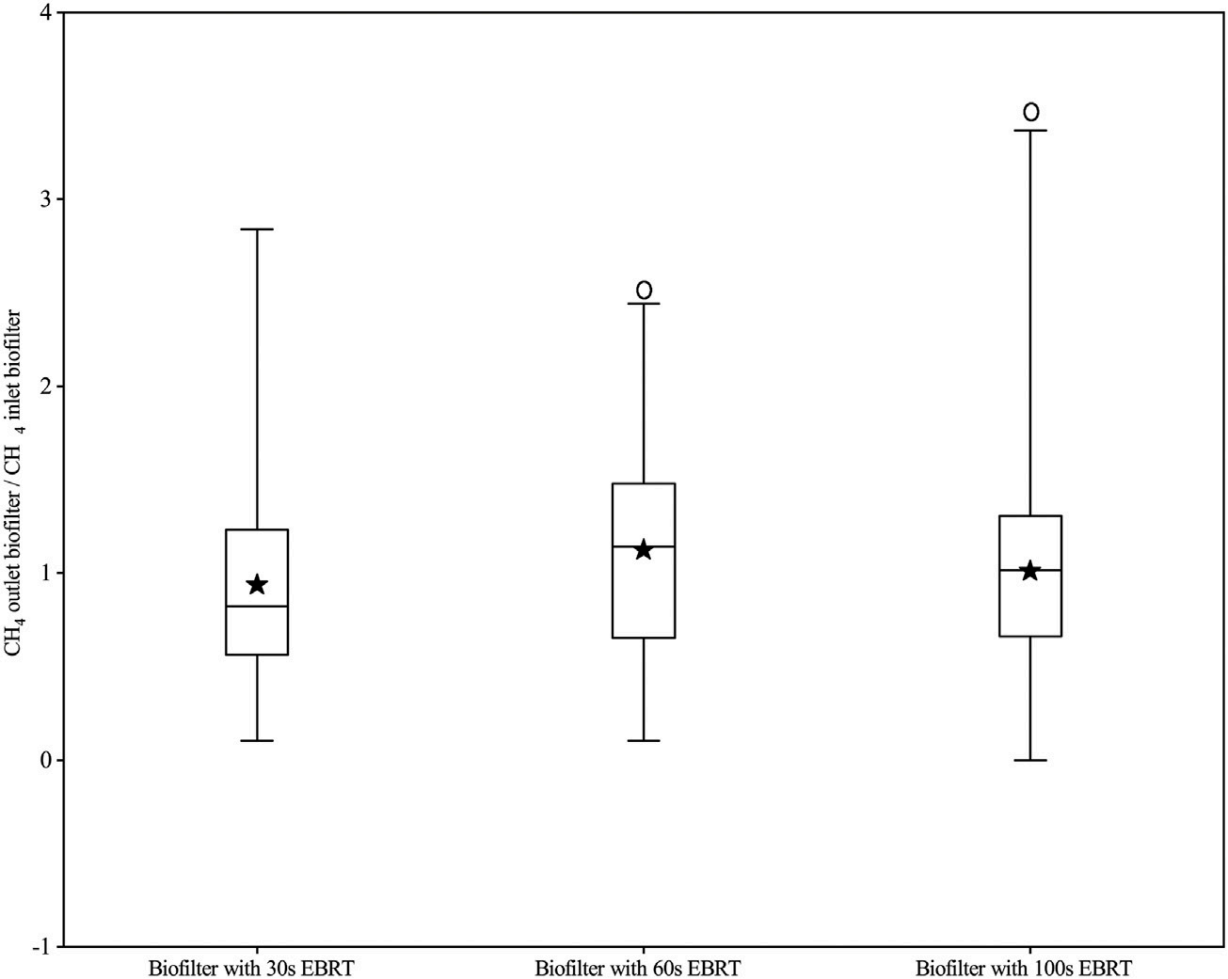
Boxplot: Ratio of CH4 concentrations of the outlet and inlet at different biofilters (Box border means 25 and 75% percentile, the solid line in the box means the median value, a solid pentacle means the mean value, a whisker means the maximum and the minimum values, and the white circle means significant differences between the inlet and outlet).

The daily mean concentrations of CH_4_ from composting ranged between 5 and 149 mg m^−3^, while the concentrations from biofilters ranged between 2 and 241 mg m^−3^. The average daily REs of CH_4_ were 6.5%, −10.5%, and −6.0% for EBRT 30, 60s, and 100s, respectively. Lim et al. (2012) reported the CH_4_ reductions ranged from 0.1% to 1.9% in biofilters with 0.3s–0.6s EBRT. Fedrizzi et al. (2018) attained nearly 100% CH_4_ 100% removal efficiencies with 756s EBRT, so enough EBRT is needed for CH_4_ removal. Akdeniz et al. (2011) achieved the removal efficiencies of 6.9%–25% for CH_4_ by using the pilot-scale biofilters with lava rock media at the high moisture levels and low inlet CH_4_ concentrations (90% moisture content and average of 31.0 ppm of inlet CH_4_ concentration), while the EBRT was just 5s. Melse and van der Werf (2005) reported that the biological conversion of CH_4_ in a biofilter is a slow process due to the low water solubility of methane (Henry’s law constant = 1.5 × 10^–3^ M atm^−1^). Biofilters with EBRT of 83–199s cannot reduce concentrations of CH_4_ emitted from municipal solid waste composting, which has been reported by Clemens and Chuls (2003). Yasuda et al. (2009) also pointed out that several microbial CH_4_ production and consumption reactions occurred, but a large portion of the CH_4_ seemed to pass straightly through the biofilter with the EBRT of 100–200s 2014; Devinny, et al., 1999). In the present study, negligible CH_4_ removal and no significant difference were found among three EBRTs, (Figure 4) and the result was similar to that of Akdeniz et al. (2011), Yasuda et al. (2009), and Lim et al. (2012). The possible reason is that the EBRT in this study is not enough for the transfer of CH_4_ from the gas phase to the biofilm phase (Melse and Van der Werf, 2005). On the other hand, although the anaerobic methanogens were not tested in this study, the retention time for anaerobic suspended growth in biofilters was about 50 days (Maia et al., 2012b); the slow acclimation of methanogens in the biofilter will lead to low CH_4_ removal efficiency.

### Global Warming Potential Emissions

Based on the measurements, the total global warming potential (GWP) emissions were calculated. Table 3 shows the GWP loading rate before and after passing through the biofilter for all the biofilters. The total GWP emission increased by 126%, 162%, and 144% for biofilters with EBRT values of 30, 60, and 100s, respectively. Significant differences were found in the total CO_2_-eq emissions from the biofilters. A lower percentage of GWP was emitted from the biofilter with 30s EBRT than in biofilters with 60 and 100s EBRT because the fraction of NH_3_-N denitrified into N_2_O-N is lower for the biofilter with 30s EBRT than that of 60 and 100s EBRT. These results showed that prolonged EBRT can improve RE of NH_3_ but increase the GWP emissions accordingly. Melse and Hol (2017) evaluated three kinds of biofiltration of exhaust air from animal houses in which the total GWP emissions increased by 60%, 45%, and 0 in biofilters with EBRT of 1.4, 2.6, and 3.3s, respectively. In their experiment, the average inlet concentrations of NH_3_ were 66, 10, and 15 ppm, respectively, and the removal efficiencies were 74%, 42%, and 38%, respectively. In the present study, the average inlet NH_3_ concentrations were 124–163 mg m^−3^, and the removal efficiencies were 82%–89%; both average inlet NH_3_ concentrations and removal efficiencies of biofilters were higher than Melse and Hol (2017).

**TABLE 3:** Cumulative nitrous oxide (N_2_O) and methane (CH_4_) emissions from biofilters used for composting exhaust ammonia (NH_3_) removal (values are means ± SE, *n* = 3)^a^.

	Biofilter inlet			Biofilter outlet		
	CH_4_ (kg.m^−3^ biofilter)	N_2_O (kg.m^−3^ biofilter)	Total GWP [kg (CO_2_ eq.) m^−3^ biofilter]^b^	CH_4_ (kg.m^−3^ biofilter)	N_2_O (kg.m^−3^ biofilter)	Total GWP [kg (CO_2_ eq.) m^−3^ biofilter]
Biofilter with EBRT 30s	3.8 (0.5)	0.52 (0.09)	240 (40)	3.2 (0.6)	1.6 (0.4)	544 (131)
Biofilter with EBRT 60s	1.9 (0.5)	0.26 (0.05)	120 (20)	2.0 (0.1)	0.9 (0.1)	314 (33)
Biofilter with EBRT 100s	1.1 (0.2)	0.16 (0.03)	72 (12)	1.1 (0.3)	0.5 (0.1)	176 (39)

a The nitrous oxide (N_2_O) and methane (CH_4_) emissions were the cumulative amount emitted from biofilters during the whole experiment which lasted for 42 days.

b GWP _CH4_ = 23 and GWP_N2O_ = 296 (Pachauri et al., 2014).

With regard to GHG emissions, it can be concluded that the lower EBRT is more suitable for biofilter systems of composting, and the parameters of the control process, such as pH, temperature, dissolved oxygen, and water content, will be useful to prevent N_2_O formation and guarantee a good NH_3_ removal efficiency.

## Conclusion

The increased N_2_O concentrations from the biofilter were strongly and positively correlated with the elimination capacities of NH_3_. The total GWP emission increased by 54%, 62%, and 61% for biofilters with 30, 60, and 100s EBRT, respectively. The total GWP emission from biofilters increases by over 50% compared to a composting system without biofilters. More NH_3_ converted into N_2_O due to higher EBRT suggested that lower EBRT is useful to prevent GHG from biofilters.

## Data Availability

The original contributions presented in the study are included in the article/Supplementary Material; further inquiries can be directed to the corresponding author.

## References

[B1] Akdeniz N. Janni K. A. (2012). Full-scale Biofilter Reduction Efficiencies Assessed Using Portable 24-hour Sampling Units. J. Air & Waste Manag. Assoc. 62, 170–182. 10.1080/10473289.2011.639479 22442933

[B2] Akdeniz N. Janni K. A. Salnikov I. A. (2011). Biofilter Performance of Pine Nuggets and Lava Rock as Media. Bioresour. Technol. 102, 4974–4980. 10.1016/j.biortech.2011.01.058 21324676

[B3] Buendia C E. Tanabe K. Kranjc A. Baasansuren J. Fukuda M. Ngarize S. (Editors) (2019). “2019Refinement to the 2006 IPCC Guidelines for National Greenhouse Gas Inventories,” in Agriculture Forestry and Other Land Use (Switzerland: IPCC), Volume 4.

[B4] Cheng H. Hu Y. (2010). Municipal Solid Waste (MSW) as a Renewable Source of Energy: Current and Future Practices in china. Bioresour. Technol. 101, 3816–3824. 10.1016/j.biortech.2010.01.040 20137912

[B5] Clemens J. Cuhls C. (2003). Greenhouse Gas Emissions from Mechanical and Biological Waste Treatment of Municipal Waste. Environ. Technol. 24, 745–754. 10.1080/09593330309385611 12868530

[B6] Devinny J. S. Deshusses M. A. Webster T. S. (1999). Biofiltration for Air Pollution Control. Boca Raton: Lewis Publishers. ISBN1-56670-289-5.

[B7] Dumont É. (2018). Impact of the Treatment of NH3 Emissions from Pig Farms on Greenhouse Gas Emissions. Quantitative Assessment from the Literature Data. New Biotechnol. 46, 31–37. 10.1016/j.nbt.2018.06.001 29909071

[B8] Dumont E. Lagadec S. Landrain P. Landrain B. Andrès Y. (2014). N_2_O Generation Resulting from Piggery Air Biofiltration. Chem. Eng. J. 248, 337–341. 10.1016/j.cej.2014.03.058

[B9] Fedrizzi F. Cabana H. Ndanga É. M. Cabral A. R. (2018). Biofiltration of Methane from Cow Barns: Effects of Climatic Conditions and Packing Bed Media Acclimatization. Waste Manag. 78, 669–676. 10.1016/j.wasman.2018.06.038 32559958

[B10] Han Z. Qi F. Wang H. Li R. Sun D. (2019). Odor Assessment of NH3 and Volatile Sulfide Compounds in a Full-Scale Municipal Sludge Aerobic Composting Plant. Bioresour. Technol. 282, 447–455. 10.1016/j.biortech.2019.03.062 30889536

[B11] Haubrichs R. Widmann R. (2006). Evaluation of Aerated Biofilter Systems for Microbial Methane Oxidation of Poor Landfill Gas. Waste Manag. 26, 408–416. 10.1016/j.wasman.2005.11.008 16386886

[B12] Huang X. Song Y. Li M. M. Li J. F. Huo Q. Cai X. H. (2012). A High-Resolution Ammonia Emission Inventory in China. Glob. Biogeochem. Cycle 26, 1–14. 10.1029/2011gb004161

[B13] Janni K. A. Jacobson L. D. Hetchler B. P. (2014). Sem-icontinuous Air Sampling versus 24-hour Bag Samples to Evaluate Biofilters on a Pig Nursery in Warm Weather. Trans. ASABE 57, 1501

[B14] Kong X. Ying S. Yang L. Xin Y. Cai Z. Zhu S. (2020). Microbial and Isotopomer Analysis of N2O Generation Pathways in Ammonia Removal Biofilters. Chemosphere 251, 126357. 10.1016/j.chemosphere.2020.126357 32146187

[B16] La H. Hettiaratchi J. P. A. Achari G. Dunfield P. F. (2018). Biofiltration of Methane. Bioresour. Technol. 268, 759–772. 10.1016/j.biortech.2018.07.043 30064899

[B17] Lim T.-T. Jin Y. Ni J.-Q. Heber A. J. (2012). Field Evaluation of Biofilters in Reducing Aerial Pollutant Emissions from a Commercial Pig Finishing Building. Biosyst. Eng. 112, 192–201. 10.1016/j.biosystemseng.2012.04.001

[B18] Liu T. Dong H. Zhu Z. Shang B. Yin F. Zhang W. (2017). Effects of Biofilter Media Depth and Moisture Content on Removal of Gases from a Swine Barn. J. Air & Waste Manag. Assoc. 67 (12), 1288–1297. 10.1080/10962247.2017.1321591 28453404

[B19] Loyon L. (2017). Overview of Manure Treatment in France. Waste Manag. 61, 516–520. 10.1016/j.wasman.2016.11.040 27955906

[B20] Maia G. D. N. Day V G. B. Gates R. S. Taraba J. L. (2012a). Ammonia Biofiltration and Nitrous Oxide Generation during the Start-Up of Gas-phase Compost Biofilters. Atmos. Environ. 46, 659–664. 10.1016/j.atmosenv.2011.10.019

[B21] Maia G. D. N. Day V G. B. Gates R. S. Taraba J. L. Coyne M. S. (2012b). Moisture Effects on Greenhouse Gases Generation in Nitrifying Gas-phase Compost Biofilters. Water Res. 46, 3023–3031. 10.1016/j.watres.2012.03.007 22465726

[B22] Melse R. W. Hol J. M. G. (2017). Biofiltration of Exhaust Air from Animal Houses: Evaluation of Removal Efficiencies and Practical Experiences with Biobeds at Three Field Sites. Biosyst. Eng. 159, 59–69. 10.1016/j.biosystemseng.2017.04.007

[B24] Melse R. W. Van der Werf A. W. (2005). Biofiltration for Mitigation of Methane Emission From Animal Husbandry. Environ. Sci. Technol. 39, 5460–5468. 10.2166/wst.2013.82610.1021/es048048q 16082981

[B25] Mudliar S. Giri B. Padoley K. Satpute D. Dixit R. Bhatt P. (2010). Bioreactors for Treatment of VOCs and Odours - A Review. J. Environ. Manag. 91, 1039–1054. 10.1016/j.jenvman.2010.01.006 20181422

[B26] Ottosen L. D. M. Juhler S. Guldberg L. B. Feilberg A. Revsbech N. P. Nielsen L. P. (2011). Regulation of Ammonia Oxidation in Biotrickling Airfilters with High Ammonium Load. Chem. Eng. J. 167, 198–205. 10.1016/j.cej.2010.12.022

[B27] Pachauri R. K. Allen M. R. Barros V. R. Broome J. Cramer W. (2014). Climate Change 2014: Synthesis Report Contribution of Working Groups I, II and III to the Fifth Assessment Report of the Intergovernmental Panel on Climate Change. Geneva, Switzerland: IPCC.

[B28] Pagans E. l. Font X. Sánchez A. (2005). Biofiltration for Ammonia Removal from Composting Exhaust Gases. Chem. Eng. J. 113, 105–110. 10.1016/j.cej.2005.03.004

[B29] Park K. J. Choi M. H. Hong J. H. (2002). Control of Composting Odor Using Biofiltration. Compost Sci. Util. 10, 356–362. 10.1080/1065657X.2002.10702098

[B30] Paulot F. Jacob D. J. Pinder R. W. Bash J. O. Travis K. Henze D. K. (2014). Ammonia Emissions in the United States, European Union, and China Derived by High-Resolution Inversion of Ammonium Wet Deposition Data: Interpretation with a New Agricultural Emissions Inventory (MASAGE_NH_3_). J. Geophys. Res. Atmos. 119, 4343–4364. 10.1002/2013jd021130

[B31] Pinder R. W. Adams P. J. Pandis S. N. (2007). Ammonia Emission Controls as a Cost-Effective Strategy for Reducing Atmospheric Particulate Matter in the Eastern United States. Environ. Sci. Technol. 41, 380–386. 10.1021/es060379a 17310695

[B32] Renner E. Wolke R. (2010). Modelling the Formation and Atmospheric Transport of Secondary Inorganic Aerosols with Special Attention to Regions with High Ammonia Emissions. Atmos. Environ. 44, 1904–1912. 10.1016/j.atmosenv.2010.02.018

[B33] Shang B. Zhou T. Tao X. Chen Y. Dong H. (2020). Simultaneous Removal of Ammonia and Volatile Organic Compounds from Composting of Dead Pigs and Manure Using Pilot-Scale Biofilter. J. Air & Waste Manag. Assoc. 71, 378–391. 10.1080/10962247.2020.1841040 33094706

[B34] Turan N. G. Akdemir A. Ergun O. N. (2009). Removal of Volatile Organic Compounds by Natural Materials during Composting of Poultry Litter. Bioresour. Technol. 100, 798–803. 10.1016/j.biortech.2008.07.010 18752939

[B35] Yang L. Kent A. D. Wang X. Funk T. L. Gates R. S. Zhang Y. (2014a). Moisture Effects on Gas-phase Biofilter Ammonia Removal Efficiency, Nitrous Oxide Generation, and Microbial Communities. J. Hazard. Mater. 271, 292–301. 10.1016/j.jhazmat.2014.01.058 24641992

[B36] Yang L. Wang X. Funk T. L. (2014b). Strong Influence of Medium pH Condition on Gas-phase Biofilter Ammonia Removal, Nitrous Oxide Generation and Microbial Communities. Bioresour. Technol. 152, 74–79. 10.1016/j.biortech.2013.10.116 24291310

[B37] Yasuda T. Kuroda K. Fukumoto Y. Hanajima D. Suzuki K. (2009). Evaluation of Full-Scale Biofilter with Rockwool Mixture Treating Ammonia Gas from Livestock Manure Composting. Bioresour. Technol. 100, 1568–1572. 10.1016/j.biortech.2008.09.033 18977137

[B38] Yasuda T. Waki M. Fukumoto Y. Hanajima D. Kuroda K. Suzuki K. (2017). Characterization of the Denitrifying Bacterial Community in a Full-Scale Rockwool Biofilter for Compost Waste-Gas Treatment. Appl. Microbiol. Biotechnol. 101, 6779–6792. 10.1007/s00253-017-8398-y 28688043

[B39] Yuan J. Du L. L. Li S. Yang F. Zhang Z. Li G. X. (2019). Use of Mature Compost as Filter Media and the Effect of Packing Depth on Hydrogen Sulfide Removal from Composting Exhaust Gases by Biofiltration. Environ. Sci. Pollut. Res. 26, 3762–3770. 10.1007/s00253-017-8398-y 30539397

[B40] Zhang H. Li G. Gu J. Wang G. Li Y. Zhang D. (2016). Influence of Aeration on Volatile Sulfur Compounds (VSCs) and NH_3_ Emissions during Aerobic Composting of Kitchen Waste. Waste Manag. 58, 369–375. 10.1016/j.wasman.2016.08.022 27595496

[B41] Zheng J. Liu J. Han S. Wang Y. Wei Y. (2020). N_2_O Emission Factors of Full-Scale Animal Manure Windrow Composting in Cold and Warm Seasons. Bioresour. Technol. 316, 123905. 10.1016/j.biortech.2020.123905 32777720

[B42] Zhu Y.-l. Zheng G.-d. Gao D. Chen T.-b. Wu F.-k. Niu M.-j. (2016). Odor Composition Analysis and Odor Indicator Selection during Sewage Sludge Composting. J. Air. Waste Manag. Assoc. 66, 930–940. 10.1080/10962247.2016.1188865 27192607 PMC5062037

[B43] Zhu-Barker X. Bailey S. K. Paw U K. T. Burger M. Horwath W. R. (2017). Greenhouse Gas Emissions from Green Waste Composting Windrow. Waste Manag. 59, 70–79. 10.1016/j.wasman.2016.10.004 27751682

